# Cancer Cells Haploinsufficient for ATM Are Sensitized to PARP Inhibitors by *MET* Inhibition

**DOI:** 10.3390/ijms23105770

**Published:** 2022-05-21

**Authors:** Concetta D’Ambrosio, Jessica Erriquez, Sonia Capellero, Simona Cignetto, Maria Alvaro, Eric Ciamporcero, Maria Flavia Di Renzo, Timothy Perera, Giorgio Valabrega, Martina Olivero

**Affiliations:** 1 Candiolo Cancer Institute, FPO-IRCCS, 10060 Candiolo, Italy; cettydambrosio@gmail.com (C.D.); jessica.erriquez@ircc.it (J.E.); sonia.capellero@ircc.it (S.C.); simona.cignetto@ircc.it (S.C.); maria.alvaro@ircc.it (M.A.); mariaflavia.direnzo@unito.it (M.F.D.R.); martina.olivero@ircc.it (M.O.); 2 Department of Oncology, University of Torino, 10129 Torino, Italy; 3 OCTIMET Oncology NV, 2340 Beerse, Belgium; eric.ciamporcero@hotmail.com (E.C.); tperera@octimet.com (T.P.)

**Keywords:** ovarian cancer, PARP inhibitor, *MET*, ATM, resistance

## Abstract

The *MET* oncogene encodes a tyrosine kinase (TK) receptor. Its activation protects cells from death but also stimulates DNA damage response by triggering excess replicative stress. Transcriptomic classification of cancer cell lines based on *MET* expression showed that response to the PARP inhibitor (PARPi) olaparib is poorer in *MET* overexpressing cell lines. Accordingly, a high *MET* expressing lung carcinoma cell line was sensitized to PARPi by *MET* TK inhibition. This was not linked solely to *MET* overexpression: other *MET* overexpressing cell lines were biochemically but not functionally responsive to combined inhibition. Moreover, exogenously induced *MET* overexpression was unable to induce resistance to PARPi. The *MET* overexpressing cell line, responsive to the combined PARP and *MET* inhibition, carried a heterozygous mutation of the ATM gene and showed an attenuated response of ATM to PARPi. Among the downstream targets of ATM activation, NuMA was phosphorylated only in response to the combined PARP and *MET* inhibition. Given the role played by NuMA in mitosis, data show that the latter is affected by *MET* and PARP inhibition in cells with haploinsufficient ATM. This is important as ATM heterozygous mutation is frequently found in human cancer and in lung carcinomas in particular.

## 1. Introduction

The treatment of cancer has changed dramatically over the last decade, driven by increased understanding of the cancer genome, leading to the development of targeted agents. Among the most successful targeted drugs, inhibitors of the poly-ADP-polymerase (PARPi) are approved for the treatment of tumors harboring defects of genes involved in repairing DNA double-strand breaks [1]. First, seminal papers introduced the concept that PARP inhibitors have synthetic lethal interaction in homologous recombination defective tumors [2,3], such as ovarian and breast cancer carrying defects of the BRCA1/2 genes. Then, given the encouraging clinical results obtained with PARPi in BRCA1/2 deficient cancers, several studies were undertaken to identify other proteins whose defects might confer PARPi sensitivity (for a review, see [4]). Among mutated genes potentially conferring susceptibility to PARPi, proteins directly involved in homologous recombination repair [5], as well as those involved in sensing DNA damage, have been associated with PARPi response/resistance [4]. Examples are REV7, RINN1/2/3, MRE11 and EZH2 (for reviews, see [4,6]). Among the latter, ataxia telangiectasia mutated (ATM) is a DNA damage checkpoint also able to indirectly activate homologous recombination repair [7] and/or to counter toxic non-homologous end-joining (NHEJ) at broken replication forks [8]. ATM is a serine-threonine kinase at the apex of a cascade responsible for global orchestration of cellular responses (for a review, see [9]). In ATM proficient cells, irradiation results in the phosphorylation of consensus sites recognized by ATM and ATR encompassing over 700 proteins [10]. Homozygous or compound heterozygous germline ATM mutation causes ataxia-telangiectasia, an autosomal recessive multisystem disorder presenting in childhood with progressive cerebellar ataxia, oculocutaneous telangiectasia, immune deficiency, radiosensitivity, and cancer predisposition [11]. In human sporadic cancers, somatic ATM aberrations have been frequently found using next-generation sequencing [12]. COSMIC reports ATM point mutations in 1% to 10% of lung, endometrial, kidney, liver, esophageal, ovarian, salivary gland, gastric, thyroid and urinary tract cancers. Excluding variants of unknown significance, 167 distinct, possibly functional, somatic mutations in ATM have been observed in a broad range of malignancies, and are associated with loss of heterozygosity in approximately 20% of cases (for a review, see [13]).

DNA damage response (DDR) is also impacted by signaling of growth factor tyrosine kinase receptors (TKRs). Several oncogenes activated in cancer lead to DDR activation as a consequence of excess replicative stress, which may result in genomic instability and alterations in checkpoint and repair mechanisms [14]. Contrarily, activation of oncogenic TKRs has been associated with resistance to genotoxic stress (see e.g., [15,16]), as it protects cells from DNA damage via different downstream signaling pathways. Remarkable advances have been made over the last two decades in the discovery and clinical development of a wide range of molecular entities targeting TKRs (for a review, see [17]). Among them, monoclonal antibodies such as Trastuzumab targeting HER2 for the treatment of breast cancer and TK small molecule inhibitors such as Gefitinib and Erlotinib targeting the epidermal growth factor receptor (EGFR) for the treatment of lung cancer have shown their clinical efficacy.

One such TKR possibly involved in the DDR is the hepatocyte growth factor (HGF) receptor, encoded by the *MET* oncogene. This oncogene was named after its isolation as an oncogenic gene from an osteosarcoma cell line transformed in vitro by the chemical carcinogen methyl-nitro-nitroso-guanidine (MNNG) [18] and found deregulated and/or activated in a variety of human tumor types and serves as an oncogenic target (for a review, see [19]). Phosphoproteomic analysis of *MET* overexpressing cells after irradiation and treatment with METi revealed the modulation of several substrates of the DDR [20]. In line with these observations, *MET* inhibition was shown to sensitize glioblastoma cells to irradiation (IR) [21]. Cooperation of *MET* inhibitors with PARPi has been reported in breast cancer cell lines exposed to hypoxia that resulted in *MET* activation [22] and nuclear transport [23] where it could phosphorylate PARP1 directly.

The aim of this work was to find a way to enlarge the spectrum of applications of approved drugs in the treatment of highly aggressive human cancers. Using in silico and in vitro experimental approaches, including functional assays and protein analyses, we show PARPi effectiveness in cells with heterozygous ATM aberration in combination with low doses of *MET* inhibitors. This work is novel and has clinical impact as low and thus manageable doses of these inhibitors in combination treatments have not been studied. In addition, we demonstrate a new mechanism for explaining the effectiveness of this drug combination.

## 2. Results

### 2.1. MET Overexpressing Cells Are Made Susceptible to PARPi by MET Inhibition

**Figure 1:**
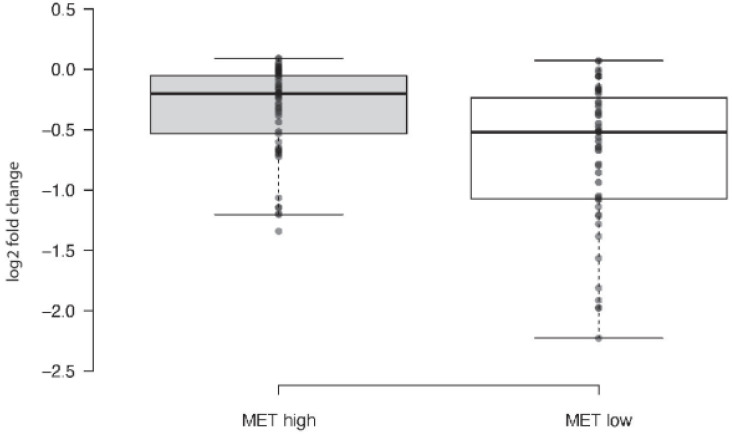
Susceptibility of a panel of 200 cancer cell lines to olaparib shown as box-plots. Log2 fold change of olaparib treated versus untreated cells is shown on the Y axis. Cell lines were subclassified based on *MET* expression according to RNAseq measured as log2 TPM+1 (transcript per kilobase million, +1). *MET* high: cell lines showing a level of expression greater than 6 log2(TPM+1). *MET* low: cell lines showing a level of expression lower than 4 log2(TPM+1). The difference between the two experimental groups was statistically significant (*p =* 0.001); *T*-test was performed using GraphPad Prism7 software.

We interrogated *MET* gene expression and susceptibility to the PARPi olaparib in 547 cancer cell lines reported in the Cancer Cell Line Encyclopedia (CCLE) project database [24], available through the tools for CCLE Data Visualization and Analysis DepMap portal (https://depmap.org/portal (accessed on 17 May 2022)). Data on olaparib susceptibility were available for 200/547 cell lines. We found that cancer cell lines overexpressing the *MET* oncogene were less sensitive to olaparib than cell lines not expressing or showing lower levels of *MET* expression (Figure 1).

**Figure 2:**
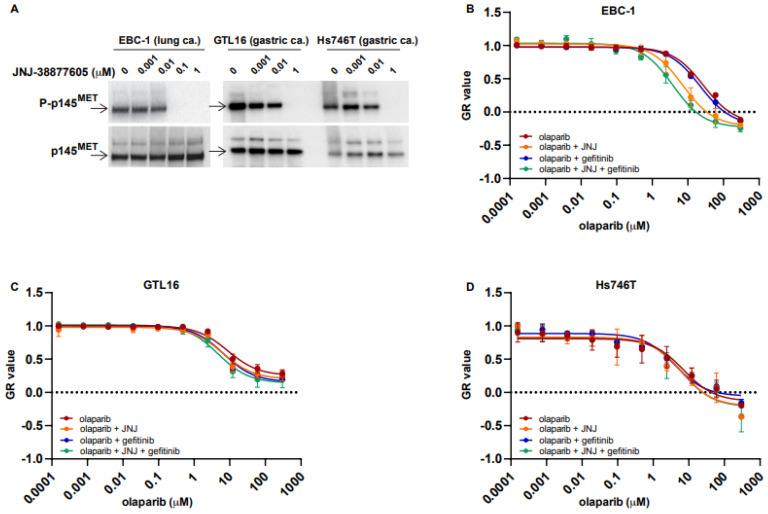
Growth rate of *MET* overexpressing cells (EBC-1, GTL16 and Hs746T) treated with the PARPi olaparib and either *MET* inhibitor JNJ-38877605 or EGFR inhibitor gefitinib. (**A**) Expression and phosphorylation status of the p145 *MET* receptor subunit in response to the indicated concentration of the inhibitor JNJ-38877605 (JNJ). (**B**–**D**) Growth rate (GR) of *MET* overexpressing cells treated with PARPi olaparib and either *MET* inhibitor JNJ-38877605 (10 nM) or EGFR inhibitor gefitinib (0.5 μM), evaluated using a 5-day crystal violet assay. Normalized growth rate (GR value) inhibition metrics have been used that take into account cell division rates across the cell lines. The sign of GR values relates directly to response phenotype: positive for partial growth inhibition, zero for complete cytostatic effect and negative for cytotoxicity. In B-D, the EBC-1 (**B**), GTL-16 (**C**) and Hs746T (**D**) cell growth rates after treatment with the indicated concentration of olaparib in combination with either JNJ or gefitinib.

Therefore, we aimed at verifying in preclinical experiments if *MET* overexpressing cells might be sensitized to olaparib by *MET* inhibition. We used the following cell lines that show the highest levels of *MET* expression [25]: EBC-1 cells established from a lung carcinoma and GTL16 cells established from a liver metastasis of gastric adenocarcinoma, both carrying *MET* amplification, and the Hs746T established from a muscle metastasis of gastric adenocarcinoma, harboring also *MET* mutation. As expected, these cells showed constitutive activation of the *MET* receptor (Figure 2A) and are the most sensitive to the highly selective *MET* kinase inhibitor JNJ-38877605 (hereafter referred to as JNJ) in the list of 923 cell lines reported in the Genomics of Drug Sensitivity in Cancer (GDSC) database (www.cancerRxgene.org (accessed on 17 May 2022)), a public resource for information on drug sensitivity in cancer cells and molecular markers of drug response [26]. In this database, the IC_50_ values for JNJ are as follows: EBC-1, 4.96 μM; Hs746T, 0.65 μM; and MKN45, 1 μM. Figure 2B–D show that these cell lines are not intrinsically susceptible to olaparib, as they all displayed a GR_50_ (normalized growth rate) [27] similar to that of lines described as olaparib resistant [28,29]. Our data are in line with the IC_50_ for olaparib reported in GDSC1, as follows: EBC-1, 45.27 μM; Hs746T, 52.16 μM; and MKN45, 111.44 μM. We then treated cells with olaparib in combination with low doses of JNJ (10 nM, i.e., 100 times lower than the IC_50_ mentioned above). The EBC-1 cells (Figure 2B), but not the GTL16 and Hs746T cells (Figure 2C,D), were more susceptible to olaparib. Notably, the JNJ dose able to sensitize EBC-1 cells to olaparib did not fully abrogate the constitutive *MET* TK phosphorylation at tyrosines 1234–1235, which is a proxy of the receptor constitutive activation (Figure 2A). We tested likewise the EGFR inhibitor gefitinib, as EGFR TK is also known to protect cells from genotoxic stress ([15]). The IC_50_ values for gefitinib reported in GDSC1 are as follows: EBC-1, 66.31 μM; Hs746T, 41.93 μM; MKN45, 75.09 μM. As shown in Figure 2B–D, none of these cell lines was affected by the combination of gefitinib and olaparib.

To further assess the possible translational impact of the cooperation between PARPi and METi, we tested this combination in a panel of ovarian cancer cell lines, using PARP inhibitors approved for the treatment of human epithelial ovarian carcinomas (EOC) that represent a fraction of susceptible ovarian carcinomas [30]. Susceptibility to PARPi was tested in the following *BRCA1/2* mutated EOC cell lines: UWB1.289 (homozygous p.2594delC *BRCA1*), IGROV1 (heterozygous frameshift deletion of *BRCA1* and missense mutation of *BRCA2*), OVCAR8 (*BRCA1* gene methylated), COV362 (splice site mutation in *BRCA1*) and, as a control, the *BRCA1/2* fully proficient A2780 cell line. These cell lines showed differential susceptibility to PARP inhibitors (olaparib, veliparib, rucaparib and niraparib, Supplementary Figure S1), in agreement with previous reports [28,29] that showed that not all *BRCA1/2* mutated lines are sensitive to PARP inhibition. Notably, in agreement with previously published data [31], the UWB1.289 cells showed the highest susceptibility to all of the PARP inhibitors tested (Supplementary Figure S1).

**Figure 3:**
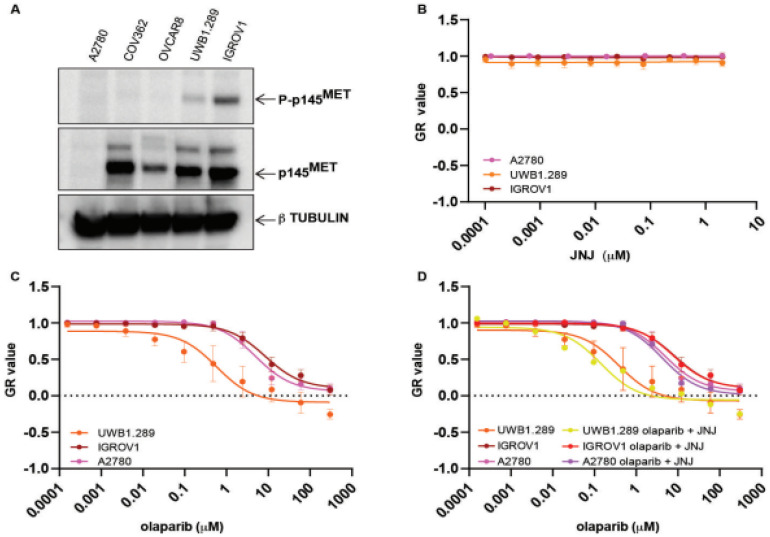
Growth rate of ovarian cancer cell lines treated with the PARP inhibitor olaparib with or without the *MET* inhibitor JNJ-38877605 (JNJ), evaluated using a 5-day crystal violet assay. Normalized growth rate (GR value) inhibition metrics have been used as described in the legend to Figure 2. (**A**) Expression (p145) and phosphorylation status (P-p145) of the 145 kDa *MET* receptor subunit in the ovarian cancer cell lines listed on the top. β tubulin was used as loading control. (**B**) Growth rate of the UWB1.289, IGROV1 and A2780 cells treated with the indicated μM concentration of the *MET* inhibitor JNJ-38877605 (JNJ). (**C**) Growth rate of the UWB1.289, IGROV1 and A2780 cells treated with the indicated μM concentration of olaparib. (**D**) Growth rate of the UWB1.289, IGROV1 and A2780 cells treated with the indicated μM concentration of olaparib with or without the *MET* inhibitor JNJ (10 nM).

Expression of the *MET* receptor was detected in all ovarian cancer cell lines except A2780 (Figure 3A). UWB1.289 and IGROV1 also showed a basal level of *MET* phosphorylation at tyrosines 1234–1235 in the absence of the added specific ligand HGF and irrespective of the level of *MET* protein expression (Figure 3A). These phospho sites represent established markers of activation of the *MET* receptor. However, we found that this activation was not indicative of susceptibility to *MET* inhibition: cells showing a basal constitutive *MET* activation were neither per se affected by JNJ (Figure 3B) nor showed a significantly increased susceptibility to olaparib when administered in combination with JNJ (Figure 3D).

### 2.2. Exogenous MET Activation Is Not Sufficient to Convert PARPi Susceptible Cells to Be Resistant

**Figure 4:**
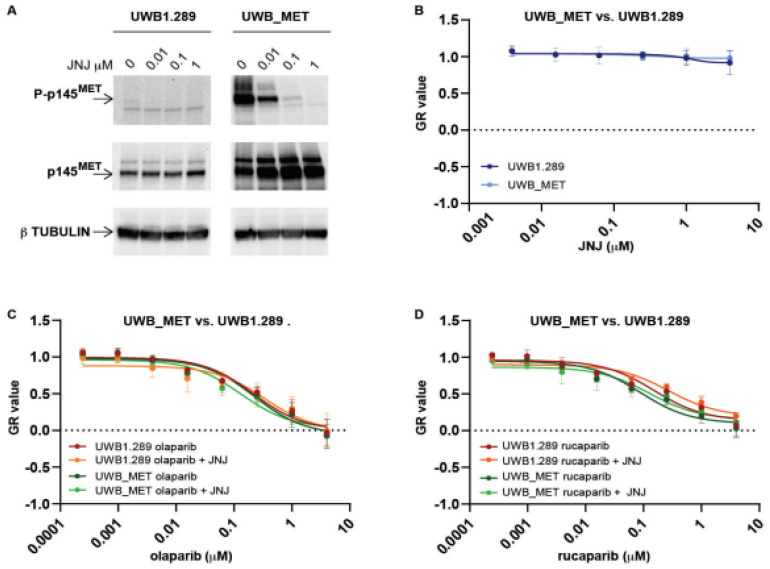
Growth rate of UWB1.289 cells transduced to overexpress the *MET* receptor (UWB_MET), treated with PARP inhibitors with or without the *MET* inhibitor JNJ-38877605 (JNJ), evaluated using a 5-day crystal violet assay. Normalized growth rate (GR value) inhibition metrics have been used as described in the legend to Figure 2. (**A**) Expression (p145) and phosphorylation status (P-p145) of the 145 kDa *MET* receptor subunit in response to the indicated μM concentration of the inhibitor JNJ-38877605 (JNJ). On the left, un-transduced UWB1.289 cells; on the right, UWB1.289 cells transduced with the wild-type *MET* transgene (UWB_MET) to overexpress the receptor. β tubulin was used as loading control. (**B**) Growth rate of the UWB1.289 and UWB_MET cells treated with the *MET* inhibitor JNJ (2 μM). (**C**,**D**) Growth rate of the UWB1.289 and UWB_MET cells treated with either the PARP inhibitor olaparib (**C**) or rucaparib (**D**) at the indicated concentrations with or without the *MET* inhibitor JNJ (2 μM).

We tested whether an increased level of *MET* expression, resulting in further *MET* activation, could impair ovarian cancer cell response to PARPi. Using lentiviral vectors, we transduced the most PARPi responsive UWB1.289 EOC cells to overexpress the wild-type *MET* receptor (Figure 4A). Exogenously induced *MET* overexpression resulted in increased constitutive activation, in the absence of the ligand, and in biochemical susceptibility to JNJ (Figure 4A). However, *MET* overexpression was not associated with either increased functional susceptibility to JNJ (Figure 4B) or increased resistance to PARP inhibitors olaparib and rucaparib (Figure 4C,D).

Altogether, these data show that the exogenously induced *MET* activation was not, on its own, sufficient to make PARPi-susceptible cells resistant to PARP inhibition.

### 2.3. In MET Overexpressing Cells with ATM Mutation, Combined PARP and MET Inhibition Affects a Downstream Cascade Leading to the Phosphorylation of the Nuclear Mitotic Apparatus (NuMA) Protein

As PARPi and METi cooperation in triple negative breast cancer cells has been previously described and reported as dependent on both proteins’ localization within cell nuclei [22] where the two proteins might interact, leading to PARP1 phosphorylation, we assessed PARP1 and *MET* localization in the above-listed *MET* overexpressing cell lines. As shown in Supplementary Figure S2, PARP1 and *MET* were respectively located in the nuclei and at the cell membrane, and were never found in the same cellular compartment.

To understand the mechanism of the distinctive response of EBC-1 cells to the combined PARPi and METi treatment, we compared their genomic profiles to those of the non-responsive cell lines and found that EBC-1 cells carry a heterozygous mutation of the *ATM* gene. 

**Figure 5:**
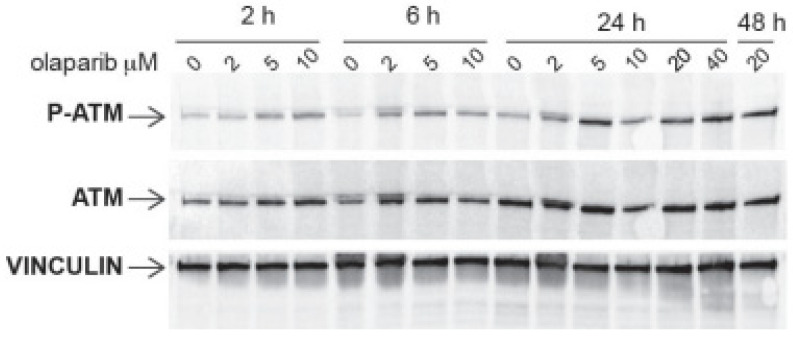
Expression (*ATM*) and phosphorylation (P-*ATM*) of the *ATM* protein in the *MET* overexpressing EBC-1 cells’ response to the treatments with the indicated concentrations of olaparib after the indicated times. Vinculin was used as loading control.

**Figure 6:**
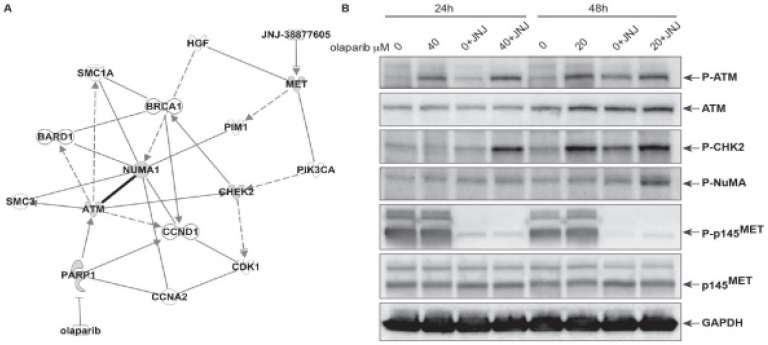
Molecules affected after treatment of the EBC-1 cells with olaparib and JNJ at the indicated concentrations and after the indicated times. (**A**) IPA allowed identifying molecules potentially affected in response to *MET* and PARP inhibition. (**B**) Western blot analysis of expression and/or phosphorylation of the indicated proteins after cell treatment at the indicated concentrations of olaparib with or without JNJ (100 nM). Proteins examined are: *ATM*, CHK2 (checkpoint kinase 2), the nuclear mitotic apparatus (NuMA) protein, *MET* and the Glyceraldehyde 3-phosphate dehydrogenase (GAPDH) used as loading control.

As reported above (Figure 2B), this genetic alteration in EBC-1 cells was not sufficient per se to make these cells responsive to olaparib. In agreement, Figure 5 and Figure 6B show that olaparib treatment was able to induce ATM phosphorylation in EBC-1 cells only at higher concentrations and at a later timepoint than those reported to be necessary for ATM activation by olaparib in non-ATM mutated cells [32,33]. The addition of JNJ to olaparib did not further increase the phosphorylation of ATM in response to olaparib alone (Figure 6B).

We then asked which mechanism might affect cell proliferation upon treatment with the combination of olaparib and JNJ. We used an unbiased approach to discover connections among molecules associated with cell response to either olaparib or JNJ. Ingenuity Pathway Analysis (IPA, Figure 6A) showed that the pathway from one inhibitor to the other involves *ATM*. It is known that *ATM* phosphorylates hundreds of substrates [10], although it is unclear which of these is functionally important. Moreover, *ATM* phosphorylates and activates other protein kinases that phosphorylate yet more substrates, meaning that *ATM*-dependent signaling events are not just restricted to factors directly phosphorylated by *ATM*. Interestingly, the unbiased IPA analysis resulted in connecting olaparib and JNJ cellular response through the *ATM* substrate CHECK2 and the nuclear mitotic apparatus (NuMA) protein. As shown in Figure 6B, we have confirmed that CHECK2 phosphorylation was associated with *ATM* activation after prolonged treatment with olaparib alone and also when combined with JNJ. Interestingly, NuMA was observed to be phosphorylated only in cells treated with the combination of olaparib and JNJ (Figure 6B). 

## 3. Discussion

After the initial biological and subsequent clinical demonstration of the effectiveness of PARPi in cells with *BRCA1/2* defects, other biomarkers of response to PARPi have been sought to select potentially responsive tumors (for a review, see [4]). We show here that heterozygous somatic *ATM* mutation alone is not sufficient to make cells susceptible to PARPi, but that heterozygous *ATM* mutation, leading to attenuated ATM activation by PARPi, makes cells more susceptible to the combined inhibition of PARP and *MET* tyrosine kinase. Indeed, *ATM* signaling attenuation has been reported to sensitize cells to DNA damaging agents, such as chemotherapeutics [34]. Moreover, lung cancer cells carrying *ATM* mutation and wild-type *RAS* and *BRAF,* such as EBC-1, display a strong dependency on MEK linked to the *ATM* heterozygous mutation [35].

As mentioned above, ATM is one of the most common genes that is somatically mutated in cancer, in particular in lung adenocarcinomas [13]. Germline heterozygous variants of *ATM* have been associated with lung cancer susceptibility and onset [36]. While *ATM* heterozygous knockout mice with 50% loss of Atm protein did not show a pronounced DNA repair phenotype [37], and *ATM* null mice were viable, mice with kinase dead *ATM* died before birth [38]. Although neither observation in mice can directly be transferred to the interpretation of somatic mutations in human cancer, this reinforces the concept that the absence of a protein does not equal the presence of a defective counterpart. *ATM* protein might be such an example: resting *ATM* forms a homodimer, and heterozygous mutations might exert dominant negative effects by interfering with the dissociation and activation associated with autophosphorylation [39]. 

*ATM* haploinsufficiency is the feature that make cells overexpressing *MET* susceptible to the combined treatment even at low doses of the small molecule METi. Data shown here confirm that *MET* expression and activation are not sufficient to make *MET* overexpressing cells, such as ovarian cancer cells, susceptible to METi or to the combined treatment, in line with the report that no synergistic effects of the combined treatment with PARPi and METi were found in cell lines with BRCA1 or BRCA2 deficiency [40]. This agrees also with the recent report that increased *MET* protein expression was found in primary cultures of PARPi resistant high-grade serous ovarian carcinomas [41]. Again, in line with data reported here, it has been recently shown that in cellular models of castration-resistant prostate cancer, *MET* inhibition enhances the efficacy of PARPi by suppressing the *ATM*/ATR and PI3K/AKT pathways [42].

We unveiled the mechanism of the efficacy of this combined PARPi and METi treatment, which relies on the failure of kinase activation triggered by *ATM*. We show here that in cells carrying heterozygous somatic *ATM* mutation, *ATM* activation by PARP1 inhibition requires higher doses and longer treatment with PARPi than those necessary for *ATM* phosphorylation in *ATM* proficient cells [32,33]. We also found that prolonged and stronger treatments were required for the substrate CHK2 phosphorylation to be observed. The mechanism by which olaparib affects the phosphorylation status of *ATM* and other molecules is still a conundrum. It is well known that olaparib causes DNA damage and that DDR results in *ATM* phosphorylation. On the other hand, it is not clear which DNA damage caused by olaparib is directly eliciting ATM phosphorylation. Indeed, PARP1 protein was initially identified for its role in the detection and repair of single-strand DNA breaks, but subsequent evidence suggested that PARP1 may also have a role in alternative DNA repair pathways, including nucleotide excision repair, nonhomologous end joining (both classical and alternative), homologous recombination, DNA mismatch repair and fork damage (for a review, see [43]).

In search of a protein involved in the specific response to PARPi and METi combination, we used IPA, which drew attention to the nuclear mitotic apparatus (NuMA) protein. This was not totally unpredictable, as the Ser395 of NuMa is one of the hundreds of consensus sequences phosphorylated by ATM in response to irradiation [10]. Moreover, the phospho-proteomic analysis of EBC-1 cells treated with irradiation and *MET* inhibition showed the increased phosphorylation of NuMA [20]. NuMA is a key structural nuclear protein, that binds microtubules and plays a role in the formation and maintenance of the spindle poles and the alignment and segregation of chromosomes during mitotic cell division (for a review, see [44]). NuMa localization and phosphorylation status vary throughout mitosis [45]. NuMA is phosphorylated by a number of different kinases, such as CDK1 [46,47] and ATM [48], and its phosphorylation is required for attachment to, and assembly of, the mitotic spindle. NuMA phosphorylation is also required for its efficient PARylation (by PARP3 and tankyarase 1), in turn necessary for correct spindle assembly [49]. It is noteworthy that olaparib, as well as rucaparib, inhibits PARP3 as well as PARP1 and PARP2 [50]. PARP3, which participates also in the DSB repair pathway(s), by interacting with NuMA and Tankyrase 1 [51], regulates mitotic progression. Altogether, our data suggest that a more complex interplay between *MET* activation/inhibition and PARP inhibitor activity might interfere with mitotic spindle organization, resulting in mitotic blockade and directing cells to mitotic catastrophe.

In conclusion, the model shown here introduces the proof of concept that *ATM* haploinsufficiency, which is found quite frequently in human cancers and alone is not able to limit the effectiveness of genotoxic pharmacological agents, such as PARP inhibitors, can be circumvented by interfering with other pathways (e.g., MET) leading to mitosis regulation. These results have clinical impact as low and thus manageable doses of these inhibitors in combination treatments are effective in killing cancer cells with a specific and identifiable genetic profile.

## 4. Materials and Methods

### 4.1. Cell Lines and Reagents

All but the GTL16 cell lines used are commercially available. The EBC-1 cells were derived from a metastatic tumor of a patient with lung squamous cell carcinoma and were purchased from the Japan Cancer Resources Bank. The other cell lines were purchased from ATCC and cultured as suggested by the provider. GTL16 was a clonal gastric cell line established in our laboratory [52]. JNJ-38877605 was from OCTIMET Oncology, and olaparib, rucaparib and gefitinib were purchased from Selleck Chemicals (Munich, Germany). Olaparib and rucaparib were used in functional assays in escalating doses, given that 10–40 μM are the effective doses reported in the literature. JNJ-38877605 and gefitinib were used at concentrations of 10 nM and 500 nM, respectively. 

### 4.2. Immunofluorescence

Cells were plated on glass coverslips coated with 0.5% gelatin in PBS. After 24 h, cells were fixed in 4% formaldehyde for 15 min at room temperature and permeabilized in PBS, 1% BSA and 0.1% Triton X-100 for 15 min at room temperature. Subsequently, cells were stained with the *MET* mouse mAb DO24 [53] and rabbit mAb anti-PARP (Cat# 9532) purchased from Cell Signaling Technology (Denver, MA, USA). Primary antibodies were revealed by Alexa Fluor 555 and 488 conjugated secondary antibodies (Thermo Fisher Scientific; Waltham, MA, USA). Cell nuclei were stained with DAPI (Thermo Fisher Scientific; Waltham, MA, USA). Confocal analysis was performed on a Leica TCS SPE and processed using ImageJ. 

### 4.3. Western Blot Analysis

For Western blot analysis, cells were plated and treated in complete medium after 24 h with the indicated drug or vehicle for the indicated doses and time points. Proteins were extracted in ice cold elution buffer (TrisHCl pH 7.4, containing EDTA, 1% Triton X-100, 10% glycerol), with protease inhibitor cocktail (1:1000), sodium orthovanadate (1:100) and phenylmethylsulfonyl fluoride (1:100), all purchased from Sigma-Aldrich (St. Louis, MI, USA), freshly added to lysis buffer. The extracted proteins were fractionated by SDS–PAGE and blotted onto nitrocellulose membrane using Trans-Blot Turbo Transfer System (BioRad, Hercules, CA, USA). The membranes were labeled overnight at 4 °C with the following antibodies: rabbit mAb anti-phosphoATM^Ser1981^ (Cat# ab81292; Abcam, Cambridge, UK), and the following antibodies, all purchased from Cell Signaling Technology (Denver, MA, USA): rabbit mAb anti-phosphoMET^Tyr1234/1235^ (Cat# 3077), rabbit mAb anti-phosphoEGFR^Tyr1068^ (Cat#3777), rabbit mAb anti-phosphoCHK2^Thr68^ (Cat#2661), rabbit mAb anti-phosphoCHK1^Ser345^ (Cat#2341), rabbit mAb anti-phosphoNuMA^Ser395^ (Cat#3429), rabbit mAb anti-NuMa (Cat#3888), rabbit mAb anti-ATM (Cat#2873), rabbit mAb anti-CHK2 (Cat#2662), mouse mAb anti-CHK1 (Cat#2360); mouse mAb anti-MET DL21 [53] and mouse mAb anti-EGFR (Cat# sc373746) purchased from Santa Cruz Biotechnology (Dallas, TX, USA). Membranes were then incubated with HRP conjugated secondary antibodies, and the chemo-luminescent signals were reveled with ECL (Thermo Fisher Scientific; Waltham, MA, USA) using the ChemiDoc Touch Imaging System (BioRad, Hercules, CA, USA).

### 4.4. CellTiter-Glo^®^ Viability Assays

CellTiter-Glo^®^ assays were performed according to the manufacturer’s protocol (Promega Madison, WI, USA) and used to evaluate the viability of cell lines after 72 h long treatment with drugs. After incubation, the luminescent signal was measured using microplate reader BioTek Synergy HTX (Winooski, VT, USA). Results were analyzed with GR metrics, which normalize cell drug response on cell doubling, and data obtained by GR analyses were plotted using GraphPad Prism 7. All experiments were performed in triplicate, each being repeated at least three times, and all data are expressed as the mean ± standard deviation. T test was performed to compare between two experimental groups using GraphPad Prism 7 software. The synergistic effects were evaluated with the combination index (CI) using the Chou–Talalay method.

### 4.5. Crystal Violet Cytotoxicity Assay

Cells were treated with the indicated drugs for 5 days and, at the end of experiments, fixed with 2% paraformaldehyde (PAF) in PBS for 40 min. After washing, cells were stained with crystal violet (10% in 20% methanol) for 40 min. Crystal violet was dissolved, incubating with 10% acetic acid for 20 min on shaker. Crystal violet absorbance signal was measured at 595-nm with the microplate reader BioTek Synergy HTX. Results were analyzed with GR metrics, which normalize cell drug response on cell doubling, and data obtained by GR analyses were plotted using GraphPad Prism 7. All experiments were performed in triplicate, each being repeated at least three times, and all data are expressed as the mean ± standard deviation. *T*-test was performed to compare between two experimental groups using GraphPad Prism 7 software.

## Supplementary Materials

The following supporting information can be downloaded at: https://www.mdpi.com/article/10.3390/ijms23105770/s1.
