# Supplementary figures and images for: Cancer Cells Haploinsufficient for ATM Are Sensitized to PARP Inhibitors by MET Inhibition
Source: Int J Mol Sci. 2022 May 21;23(10):5770. doi: 10.3390/ijms23105770 (PMC9146142; doi:10.3390/ijms23105770)

Supplementary Figure S1

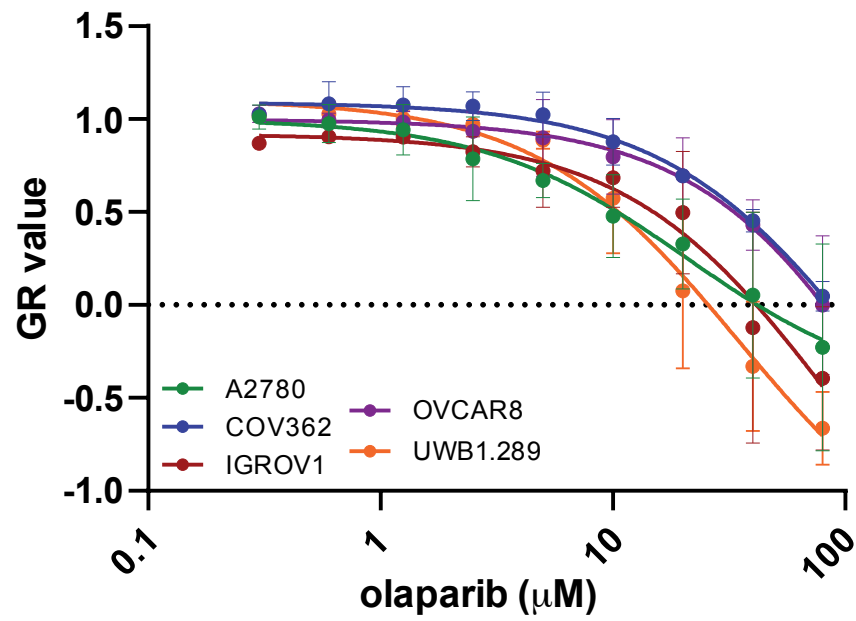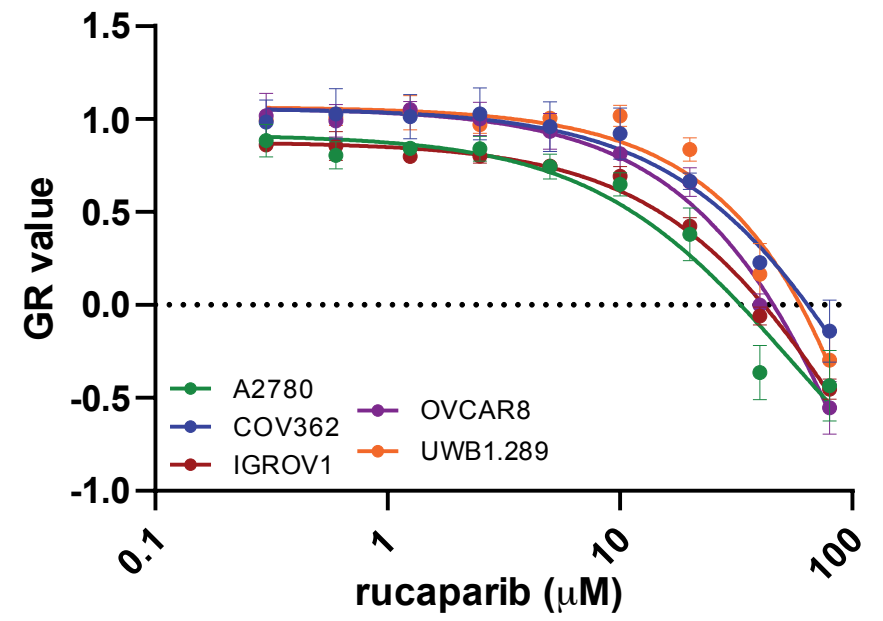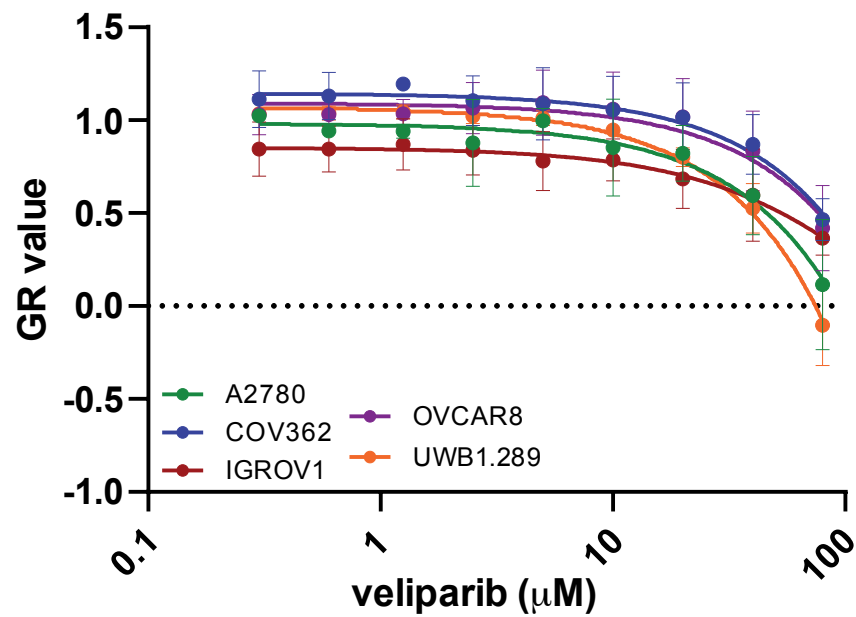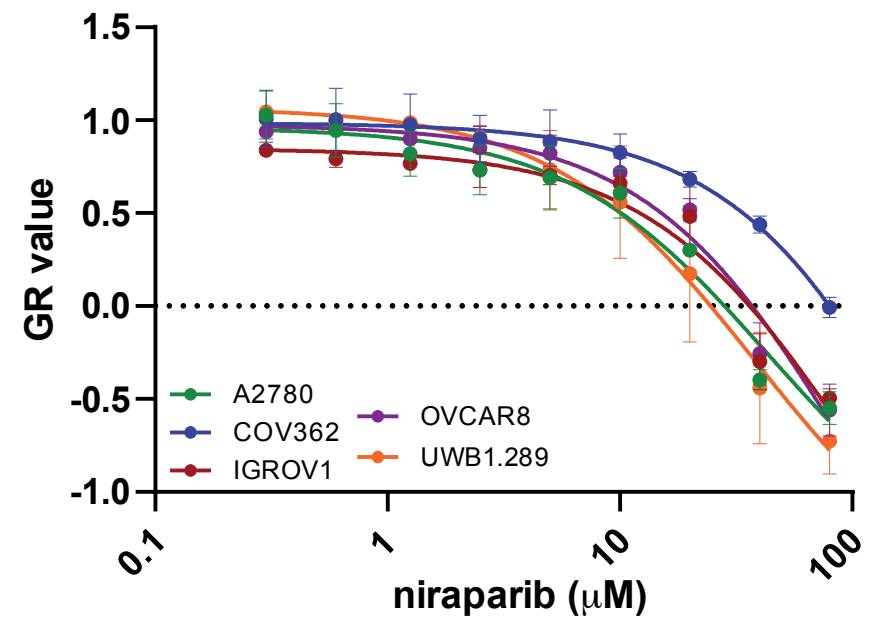

Supplementary Figure S2

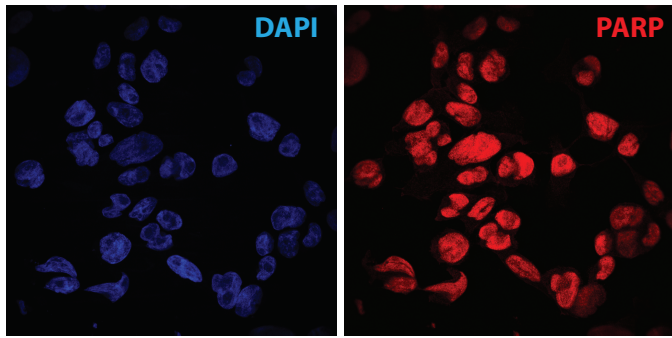

EBC-1

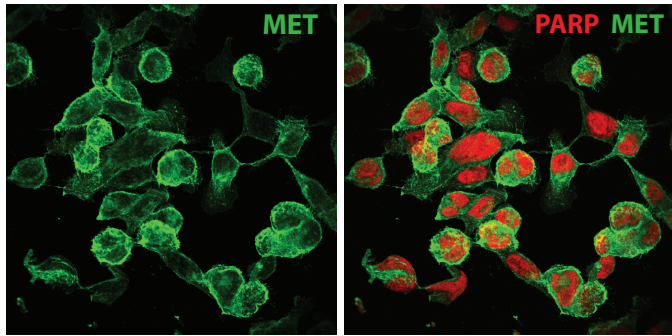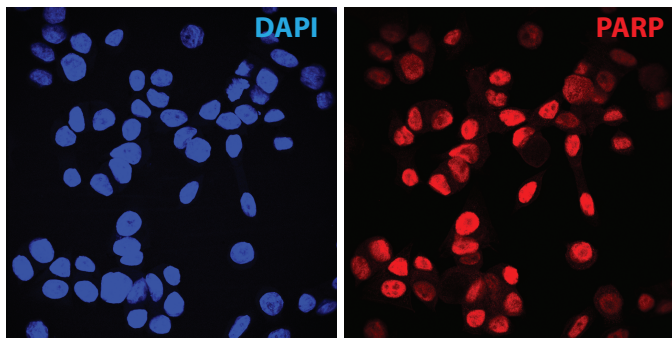

GTL16

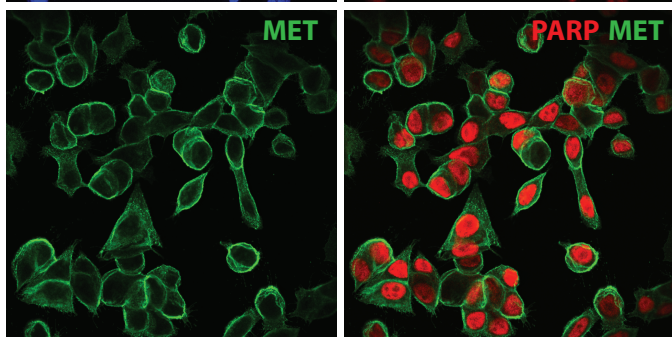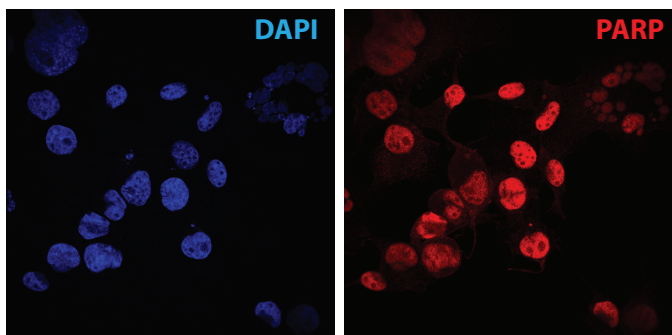

Hs746T

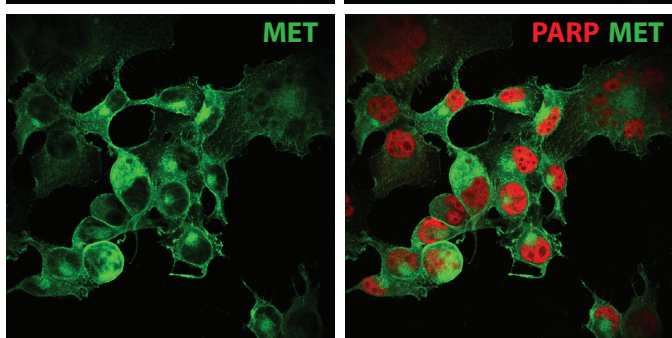

Supplement: Supplementary file 1 [file ijms-23-05770-s001.zip › ijms-1675110-supplementary.pdf]
